# The Multifaceted Impact of Environmental Pollutants on Health and Ecosystems

**DOI:** 10.3390/biom14081021

**Published:** 2024-08-17

**Authors:** Marina Piscopo, Carmela Marinaro, Gennaro Lettieri

**Affiliations:** Department of Biology, University of Naples Federico II, Via Cinthia, 21, 80126 Naples, Italy

Environmental pollutants have pervasive and far-reaching effects on both ecosystems and human health. Recent scientific studies provide crucial insights into how various contaminants—ranging from heavy metals, such as nickel, mercury, and chromium, to microplastics—impact biological systems. In addition, agricultural practices significantly contribute to the dissemination of ARGs (antibiotic resistance genes) in surface waters, posing risks to human health. This editorial synthesizes findings from multiple studies, seven research articles, and one review article to highlight the intricate mechanisms and broader implications of these pollutants [1,2,3,4,5,6,7,8].

## 1. Nickel and Marine Life: A Silent Threat

Nickel exposure has a significant effect on the reproductive health of marine organisms, as shown in a study on *Mytilus galloprovincialis*. Researchers found that exposure to nickel chloride (NiCl_2_) is able to change the properties of protamine-like proteins (PLs), the major basic nuclear protein components of the sperm chromatin of this organism. In particular, nickel affects these proteins and how they bind to DNA, thereby altering the sperm chromatin structure. These alterations were observed at various NiCl_2_ concentrations (5, 15, and 35 µM), with notable increases in MNase accessibility and PARP expression, indicating cellular stress and potential reproductive toxicity. These findings underscore the complex molecular mechanisms by which nickel exerts its deleterious effects on marine reproductive health [1].

## 2. Nickel's Impact on Human Health: Beyond the Environment

Nickel also poses significant risks to human health, particularly ocular health. A study on human corneal epithelial cells (HCECs) demonstrated that nickel exposure leads to oxidative damage and apoptosis. Nickel induced a concentration-dependent decrease in cell viability and increased reactive oxygen species (ROSs) production, which was associated with upregulation of apoptotic genes. These findings suggest that nickel pollution could have serious implications for ocular health, particularly for individuals exposed to nickel-containing dust and particulate matter [4].

## 3. Chromium (VI) Exposure Affects Reproductive Health of Marine Organisms

Another heavy metal with significant reproductive toxicity is hexavalent chromium. In *Mytilus galloprovincialis*, exposure to nanomolar chromium (VI) doses also resulted in changes in protamine-like proteins and their DNA-binding properties, as well as in the expression of genes related to stress and PL proteins. This study found that even low levels of chromium(VI) can alter sperm chromatin structure and affect reproductive health, highlighting the potential toxicity of this pollutant [2].

## 4. Microplastics: Invisible yet Potent Pollutants

Microplastics (MPs) are emerging as a significant environmental pollutant with adverse effects on human health. Research on polystyrene microplastics (PS-MPs) has shown that they induce pro-inflammatory and cytotoxic responses in human and mouse intestinal cell lines. Very recently, microplastics have also been found in human semen [9]. Increased oxidative stress, inflammation, and cytokine release were observed following exposure to PS-MPs, highlighting their potential to cause gastrointestinal problems. This study highlights the need for strategies to reduce plastic pollution, adding to the growing body of evidence on the harmful effects of MPs [7].

## 5. Mercury's Effects on Reproductive Health in Marine Organisms

Mercury is one of the most dangerous environmental pollutants. In addition to causing a range of health effects in humans, including changes in hemoglobin and red blood cell membrane proteins [10], this metal is also responsible for a range of changes in marine organisms. In *Mytilus galloprovincialis*, mercury exposure caused significant changes in gonadal morphology, stress gene expression, and sperm chromatin structure [11,12]. These alterations suggest that mercury can severely impact reproductive health in marine species, underscoring the importance of monitoring and reducing mercury pollution in aquatic environments [6].

## 6. Endocrine Disruptors and Arthritis Progression

Endocrine disruptors (EDs) are chemicals that interfere with hormonal regulation and can also exacerbate conditions such as arthritis. Research on collagen-induced arthritis in mice revealed that exposure to various EDs worsened clinical symptoms, increased inflammation, and caused oxidative damage. These findings suggest that EDs can significantly impact bone and joint health, further emphasizing the need to limit exposure to these chemicals [8,13,14].

## 7. The Spread of Antibiotic Resistance: A Growing Concern

Another very important aspect is that agricultural practices are a major contributor to the spread of ARGs (antibiotic resistance genes) in surface waters, with consequent risks to human health. The prevalence of ARGs, particularly those related to sulfonamide antibiotics, in waterbodies surrounding pastures and greenhouses was demonstrated in a comprehensive study conducted in the Netherlands. The study suggested that larger water bodies or green buffer zones could mitigate the impact of horizontal gene transfer on the spread of ARGs. To control the spread of antibiotic resistance from agricultural sources, these results underline the need for effective management strategies [3,15,16,17].

## 8. Environmental and Genetic Interactions in Reproduction

Understanding how environmental and genetic factors interact is critical to understanding reproductive health. Semen is now regarded as an early indicator of the health of the environment and of the reproductive and general health of an organism [18]. Sperm quality was also considered a potential indicator of susceptibility to SARS-CoV-2 in polluted areas, and a possible dangerous synergy between air pollution and COVID-19 was observed [19,20]. Spermatozoa also possess great plasticity to adapt to changing environmental conditions, as demonstrated in *Mytilus galloprovincialis* under hyposalinity conditions [21,22,23,24]. Recent research has highlighted how, through genetic and epigenetic changes, environmental agents can affect male fertility and the health of offspring. Moreover, some heavy metals exhibit seasonally dependent behavior, as demonstrated for cadmium [25]. In addition, the mixture of some heavy metals is more dangerous than individual metals [26]. The gonads and spermatozoa of *Mytilus galloprovincialis* respond to certain pollutants by also altering the type and levels of metabolites [27,28]. These findings provide a comprehensive perspective on how environmental contaminants can affect reproductive outcomes across generations. They highlight the need for further research and protective measures.

## 9. Broader Implications: From Sperm to Offspring

A review of how environmental pollutants affect reproductive outcomes further explores the complex relationship between environmental pollutants and genetic factors. The dynamic nature of the epigenetic landscape means that environmental perturbations can have long-lasting effects on the fertility and health of the offspring [29,30]. After all, epigenetic modifications of chromatin are known in various diseases [31,32,33,34,35]. This review brings together the current body of research to show how paternal environmental interactions can reverberate through the generations, affecting not only immediate health outcomes but also long-term genetic and epigenetic profiles. 

## 10. Call to Action

These studies underscore the need for holistic approaches to environmental protection, highlighting the multifaceted effects of environmental pollutants on reproductive health. To protect both the environment and human health, it is essential to strengthen regulations on pollutants, invest in cleaner technologies, and promote sustainable practices. Only through such a comprehensive effort can we hope to address the profound challenges posed by environmental pollutants.

## 11. Conclusions

The studies reviewed in this editorial are evidence of the widespread and diverse impacts of environmental pollutants on health and ecosystems. From marine life to human health, the evidence urgently requires comprehensive risk reduction strategies. Key steps to protect both the environment and public health include reducing exposure to heavy metals, controlling the spread of antibiotic resistance, and addressing the proliferation of microplastics and endocrine disruptors.
